# Effects of a Nurse-Coordinated Transitional Care Service on Self-Management, Functional Status, Psychological, and Patient-Centered Outcomes in Patients with COPD: A Randomized Controlled Trial

**DOI:** 10.3390/healthcare14101365

**Published:** 2026-05-15

**Authors:** Su Kyoung Kim, Deog Kyeom Kim, Yukyung Park, Woo Jin Kim, Seon-Sook Han, Yeon Jeong Heo, Da Hye Moon, Oh Beom Kwon, Myung Goo Lee, Ji Young Hong, Jung-Kyu Lee, Eun Young Heo, Hyun Woo Lee, Yu-Seong Hwang, Chang Youl Lee, Heui Sug Jo

**Affiliations:** 1 Department of Health Policy and Management, School of Medicine, Kangwon National University, Chuncheon 24341, Republic of Korea; tnrud5698@kangwon.ac.kr (S.K.K.); yukyungpark@gmail.com (Y.P.); hys1077@gmail.com (Y.-S.H.); 2 Division of Pulmonary and Critical Care Medicine, Department of Internal Medicine, Seoul Metropolitan Government-Seoul National University Boramae Medical Center, Seoul 07061, Republic of Korea; kimdkmd@snu.ac.kr (D.K.K.); jk1909@empas.com (J.-K.L.); eunyoungheo@gmail.com (E.Y.H.); athrunzara86@naver.com (H.W.L.); 3 Department of Preventive Medicine, Kangwon National University Hospital, Chuncheon 24289, Republic of Korea; 4 Department of Internal Medicine, Kangwon National University Hospital, Chuncheon 24289, Republic of Korea; pulmo2@kangwon.ac.kr (W.J.K.); ssunimd@kangwon.ac.kr (S.-S.H.); yonjong1954@naver.com (Y.J.H.); ansekgo@naver.com (D.H.M.); shob86@naver.com (O.B.K.); 5 Division of Pulmonary, Allergy and Critical Care Medicine, Department of Internal Medicine, Chuncheon Sacred Heart Hospital, Hallym University College of Medicine, Chuncheon 24253, Republic of Korea; mglee@hallym.or.kr (M.G.L.); mdhong@hallym.or.kr (J.Y.H.)

**Keywords:** patient centeredness, chronic obstructive pulmonary disease, coordinator, transitional care

## Abstract

Background: Transitioning from hospital to home presents substantial challenges for patients with chronic obstructive pulmonary disease (COPD), often leading to difficulties maintaining self-management, functional independence, and psychological well-being after discharge. Although transitional care programs are increasingly implemented, their effects on multidimensional patient-centered outcomes remain insufficiently examined. This study aimed to evaluate the effectiveness of a nurse-coordinated transitional care service for patients with COPD during the transition from hospital to home and to examine its broader implications for improving continuity of care and patient-centered outcomes within the healthcare system. Methods: This randomized controlled trial was conducted in three university hospitals in South Korea between November 2022 and December 2024. A total of 465 patients were randomly assigned to either a nurse-coordinated transitional care intervention group or a usual care group. The intervention included structured self-management education during hospitalization, post-discharge home visits, and follow-up telephone consultations during the first month after discharge. Outcomes were assessed at baseline, 1 month, and 3 months. Statistical analyses included linear mixed-effects models for continuous outcomes and chi-square tests and independent t-tests for group comparisons. Results: Patients in the Transitional Care Group (TCG) showed marked improvements: disease awareness increased from 27.9% to 94.3% (vs. 35.7% in the Usual Care Group [UCG], RR = 2.64, 95% CI: 2.19–3.18, *p* < 0.001) and exercise adherence to 76.3% (vs. 43.0%, RR = 1.78, 95% CI: 1.49–2.11, *p* < 0.001). After adjusting for age, cognitive function declined in both groups but showed significantly smaller decreases in the TCG than in the UCG at 3 months (mean difference = −0.92, *p* < 0.001), and IADL demonstrated significantly better preservation in the TCG (mean difference = −1.77, *p* < 0.001). Self-efficacy declined in both groups but remained significantly higher in the TCG (mean difference = 2.65, *p* < 0.001). Anxiety and depression were significantly reduced in the TCG compared with the UCG (anxiety: −1.45, *p* < 0.001; depression: −2.72, *p* < 0.001). After adjusting for age, discharge preparedness and post-discharge management capacity were significantly higher in the TCG than in the UCG (adjusted mean differences = 3.25 and 4.93, respectively; both *p* < 0.001). Conclusions: These findings indicate that nurse-coordinated transitional care enhances patients’ self-management capacity and improves patient-centered outcomes during the transition from hospital to home.

## 1. Introduction

Chronic obstructive pulmonary disease (COPD) is characterized by airway obstruction and persistent respiratory symptoms caused by exposure to harmful particles or gases that increase airway resistance and damage lung tissue [1]. Globally, COPD has high morbidity and mortality rates and was the fourth leading cause of death worldwide in 2021 [2].

Transitioning from hospital to home is a pivotal phase for patients, requiring them to manage their conditions independently. Previous research has revealed that 20% of hospitalized patients with COPD are readmitted within 30 days or encounter post-discharge complications due to insufficient preparation for inhaler use and self-management [3,4,5,6]. To address these challenges, transitional care services (TCSs) have been introduced as post-discharge support. TCSs adopt a patient-centered care model designed to improve the quality of life and treatment outcomes of patients with chronic diseases and their families. Nurses or multidisciplinary teams conduct comprehensive assessments during hospitalization, develop tailored care plans, and provide targeted disease management education. Furthermore, TCSs seamlessly integrate social welfare services and follow-up care, such as home visits and teleconsultations, to bolster patients’ disease management capabilities and facilitate smoother recovery [7]. Previous studies have highlighted the effectiveness of TCSs in reducing dyspnea [8], enhancing quality of life [9], and alleviating depression [10] in patients with COPD.

Effective communication between healthcare teams and patients during hospitalization and transitional periods is critical [11]. Patient-centered communication involves simplifying information about diseases, treatments, and potential side effects to enhance patient understanding [12] or engaging patients in decision-making processes that align with their values and concerns [13,14,15,16]. Nurses who frequently interact with hospital patients are key members of transitional care teams. Effective patient-centered communication by nurses fosters trust and contributes to improved patient outcomes [17,18,19]. The communication skills of nurses are essential for planning care transitions and educating patients from the time of admission [20,21]. Additionally, during the transition period, nurses play a pivotal role in coordinating with primary physicians to adjust medication regimens and arrange follow-up visits. They also monitor patients and provide necessary follow-up actions [22]. Federman et al. [23] reported that effective communication between nurses and chronically ill patients regarding their medications was associated with shorter hospital stays, reduced readmission rates, and fewer emergency room visits. Møller et al. [24] emphasized the importance of providing training to strengthen the communication and counseling competencies of nurses who deliver TCSs, especially for elderly patients with multiple chronic conditions. Similarly, Coleman and Boult [20] highlighted the need for efficient communication training among multidisciplinary team members, including nurses, physicians, and social workers, to ensure the effective delivery of TCSs. Despite this growing evidence, prior studies have primarily focused on communication processes or service utilization outcomes, with limited attention to comprehensive evaluation of the impact of nurse-coordinated transitional care on multidimensional patient-centered outcomes. Moreover, although nurses are central to transitional care delivery, their specific contribution as coordinators of care transitions has not been sufficiently examined within an integrated outcome framework.

Accordingly, the present study systematically aligned the six core components of the Chronic Care Model with TCSs in designing the intervention, with nurses assuming a central role in providing self-management support and coordinating care transitions from hospitalization through the post-discharge period. Outcome measures were derived using an indicator framework developed through a Delphi process based on the UK National Health Service (NHS) Outcomes Framework [25]. Reflecting the growing emphasis on patient-reported outcomes as a means of evaluating intervention effectiveness from the patient’s perspective, this study sought to comprehensively assess the effects of nurse-coordinated transitional care interventions using a multidimensional approach rather than a single outcome indicator. Accordingly, outcome measures comprised health-related self-management behaviors indicators (disease awareness, smoking status, and exercise adherence), functional status indicators (cognitive function, and instrumental activities of daily living), psychological well-being indicators (self-efficacy, anxiety, and depression), and patient-centered indicators (discharge preparedness, post-discharge self-management capacity, and patient satisfaction). In line with this approach, this study aimed to evaluate the effectiveness of a nurse-coordinated transitional care service and to examine its broader implications for improving continuity of care and informing the development of integrated, patient-centered healthcare systems.

## 2. Materials and Methods

### 2.1. Ethics Statement

The study was conducted in accordance with the ethical principles of the Declaration of Helsinki. The study protocol was approved by the institutional review boards of Kangwon National University Hospital (IRB number KNUH-B-2022-08-003-003), Hallym University Chuncheon Sacred Heart Hospital (IRB number CHUNCHEON IRB 2022-07-002-001), and Seoul National University Boramae Medical Center (IRB number 20-2023-91). This study was registered with the Clinical Research Information Service (CRIS; https://cris.nih.go.kr accessed on 13 May 2026) under the clinical trial number KCT0007937, and the registration date was 24 November 2022. Participants were prospectively recruited between 28 November 2022 and 31 December 2024. All participants were adults and provided written informed consent to participate, and all consent forms were obtained and archived by trained nurses. No minors were included in this study. The reporting of this randomized controlled trial adheres to the CONSORT 2025 guidelines, and the CONSORT checklist is provided in Supplementary File S1.

### 2.2. Sample Size Calculation

The sample size calculation was based on prior institutional data, indicating a 1-month all-cause readmission rate of 26.2% among patients with COPD. For this trial, we assumed that the intervention would reduce the readmission rate to 15.6% [26]. A one-sided Z-test was applied under the assumption that the intervention would not increase readmission risk, with 80% power and a significance level of 0.05. Accordingly, the required sample size was estimated to be 362. To account for potential attrition, approximately 28.5% additional participants were recruited, resulting in a total enrollment of 465 to ensure that 362 participants remained for the final analysis. Clinical outcomes, such as readmission rates derived from this sample, are reported in a separate manuscript that is currently under review.

### 2.3. Randomization

Randomization was performed by the clinical coordinator, who assigned participants to the transitional care group (TCG) or usual care group (UCG) in the order of hospital admission using a computer-generated random number sequence. To ensure balance in disease severity between groups, participants were stratified based on FEV1 (≥50% vs. <50%) prior to randomization. Allocation concealment was maintained by sequentially enrolling patients according to this pre-generated list without prior disclosure of group assignment to the research team. Participants remained blinded to their group assignment after randomization, and all data collection and study procedures were conducted under the supervision of a Clinical Research Associate.

### 2.4. Study Design and Participants

To evaluate the effectiveness of nurse-coordinated, patient-centered TCSs, participants were recruited from patients with COPD hospitalized at three university hospitals in South Korea: Kangwon National University Hospital, Hallym University Chuncheon Sacred Heart Hospital, and Seoul Boramae Medical Center. TCSs were administered from the time of hospital admission to three months after discharge.

The inclusion criteria were (1) diagnosis of COPD (ICD codes J44.0, J44.9) as a primary or secondary condition based on spirometry results (FEV1/FVC < 0.7) in the electronic medical records and (2) written consent to participate in the study. The exclusion criteria were as follows: (1) current treatment for cancer, (2) mental or behavioral disorders that could impede participation (survey and education compliance), and (3) dementia. Participants were stratified based on their most recent spirometry results, as recorded in their electronic medical records, and were randomly assigned. To ensure comparable clinical severity between TCG and UCG, the participants were categorized into mild (FEV1 ≥ 50%) and severe (FEV1 < 50%) groups, followed by randomization within each stratum. This stratified randomization approach was used to control for potential differences in disease severity between groups at baseline. Random allocation was performed using a random number table, and allocation concealment was maintained by limiting access to the randomization sequence exclusively to the research nurse responsible for participant assignment. The trial was conducted in a single-blind manner; participants were blinded to their group allocation, but the research team, including the nurse in charge of the allocation, was aware of the assignments.

**Figure 1:**
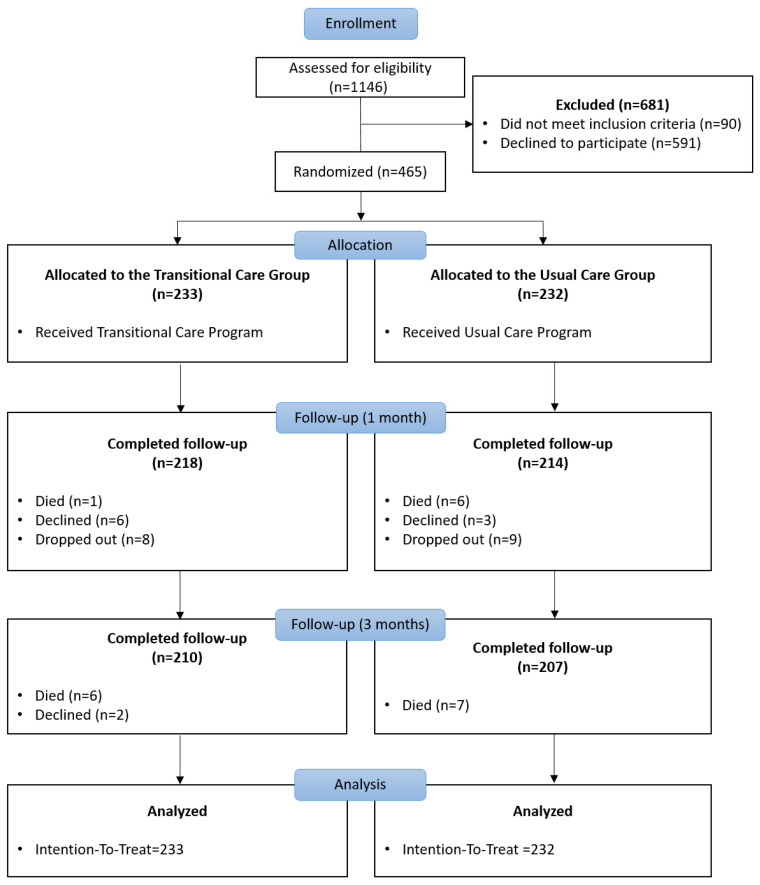
CONSORT flow diagram. The flow diagram illustrates the study procedure.

A total of 465 patients were recruited (TCG, 233; UCG, 232). However, 48 participants were excluded from the study due to death, withdrawal of consent, cancer diagnosis, or other reasons. Finally, 417 patients (TCG, 210; UCG, 207) were included in this study (Figure 1).

### 2.5. Intervention (Patient-Centered Transitional Care Service)

The transitional care service in this study was systematically designed to correspond to the six core components of the Chronic Care Model.

First, self-management support was implemented through structured self-management education provided during hospitalization, followed by home visits and telephone monitoring after discharge. A total of two nurses from each participating hospital were assigned to deliver the intervention, all of whom were nurses specifically hired for the study rather than regular hospital staff. Nurses were eligible if they had completed a four-year nursing degree and had undergone standardized training based on an educational curriculum developed by the research team prior to the intervention. To ensure competency, all nurses completed the training program before delivering the intervention, and a standardized intervention protocol and educational materials were used to minimize inter-interventionist variability.

The training program included knowledge and understanding of COPD and the transitional care model (3 h), use of oxygen and inhalants (1 h), and home visits (1 h), as well as COPD-specific clinical skills (e.g., inhaler techniques and pursed-lip breathing) and communication and coaching skills, including active listening and verbal and nonverbal communication techniques to enhance patient motivation (2 h). Each patient was assigned to a designated nurse who provided continuous care from hospitalization through the post-discharge period. During hospitalization, self-management education was delivered through face-to-face sessions using printed educational materials, with each session lasting approximately 20 min. The educational materials were based on the evidence-based The Montreal Chest Institute’s Living Well with COPD (LWWCOPD) program and were adapted and translated to fit the context of this study. The materials consisted of 10 modules covering key aspects of COPD self-management, including disease understanding, symptom recognition, inhaler use, breathing techniques, daily management, health promotion, and symptom monitoring through management diaries. After discharge, home visits were conducted by nurses at patients’ residences using the same materials, with each visit lasting approximately 20 min, and telephone monitoring sessions of approximately 15 min were implemented to reinforce self-management and monitor patients’ health status. Through this preparation, nurses delivered tailored education that reflected patients’ levels of disease understanding and individual preferences, and promoted sustained improvement in post-discharge self-management capacity through repeated feedback and positive reinforcement.

Second, delivery system design was operationalized through a standardized care pathway in which nurses coordinated the entire transitional period from hospitalization to post-discharge. Specifically, within 72 h of hospital admission, a pulmonologist assessed patients’ clinical eligibility, and eligible patients were enrolled and referred to the transitional care nurse. Patients received two sessions of structured self-management education during hospitalization. The nurse then established a trusting relationship with the patient through empathic communication and active listening, followed by an initial comprehensive assessment. Issues identified during this assessment—including difficulties with inhaler use, experiences of medication side effects, medication adherence, and the need for social welfare services—were shared during multidisciplinary meetings and used to inform subsequent adjustments to treatment and care plans.

Crucially, the transitional care nurse acted as a key clinical liaison, bridging the gap between hospital-based specialists and the patient’s home environment.

Beyond delivering education, the nurse proactively coordinated care by feeding back real-world barriers identified during home visits—such as challenges in medication management, environmental risks, and unmet social needs—to the medical team.

This bidirectional communication facilitated timely medication adjustments, supported shared clinical decision-making, and enabled linkage to appropriate community resources. Through this hub role, the nurse ensured seamless continuity of care, transforming what is often a fragmented discharge process into a cohesive and patient-centered management plan.

Third, decision support was implemented by providing situation-specific action plans that guided patients on when to seek outpatient care or utilize medical services in response to symptoms of acute COPD exacerbation. Nurses supported patients’ symptom appraisal and medical decision-making through a first home visit conducted within 48 h after discharge, a second home visit at one month post-discharge, and weekly telephone monitoring during the first month following discharge.

Fourth, clinical information systems were applied using a clinical research management system developed by the Korea National Institute of Health to ensure continuity and efficiency in healthcare delivery. This system enabled systematic documentation of initial assessment findings and follow-up monitoring data, ensuring consistent use of patient information throughout the intervention process.

Fifth, healthcare organization was addressed through the implementation of hospital-level transitional care protocols [27], organizational support for nurse-led interventions, and regular multidisciplinary meetings involving pulmonologists and social workers.

Finally, community resources were integrated through linkage to social welfare services during hospitalization and after discharge. During post-discharge home visits, nurses assessed housing conditions, ventilation status, and fall risk factors, thereby supporting patients’ ability to live safely and independently within the community.

**Figure 2:**
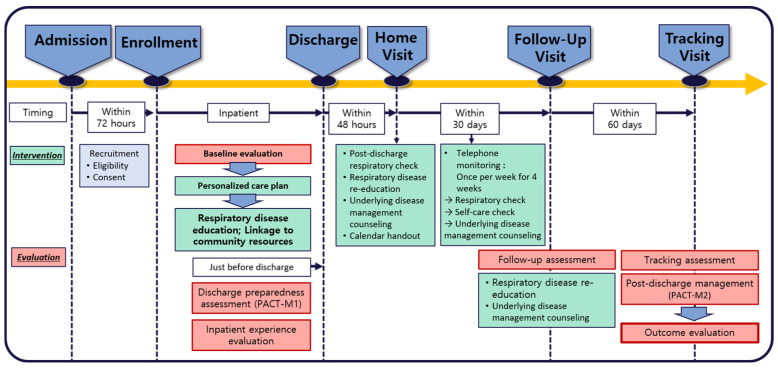
Flow Diagram of the Study Implementation Phases. The diagram illustrates how the TCS intervention is delivered at each stage of the study.

In the UCG, the participants were provided with standard health management information at the time of discharge. They received guidance on smoking cessation, medication adherence, respiratory rehabilitation, and inhaler management. However, nurse-coordinated education was not provided. The detailed intervention protocols are shown in Figure 2.

### 2.6. Instruments

This study collected sociodemographic characteristics and assessed outcomes using a structured framework reflecting key domains of nurse-coordinated transitional care. Outcome measures were organized into three domains: health-related self-management behaviors indicators (disease awareness, smoking status, and exercise adherence), functional status indicators (cognitive function, and instrumental activities of daily living), psychological well-being outcomes (self-efficacy and hospital anxiety and depression), and patient-centeredness (Partners at Care Transitions Measure [PACT-M1 and PACT-M2] and patient satisfaction).

This domain-based organization was designed to capture the multidimensional effects of nurse-coordinated transitional care, particularly patient-reported outcomes that are sensitive to care coordination during the transition from hospital to home.

#### 2.6.1. Health-Related Self-Management Behaviors Outcomes

- Disease awareness

Disease awareness was defined as whether patients could accurately identify their diagnosis as chronic obstructive pulmonary disease (COPD). Accordingly, it was assessed as a dichotomous variable (0 = no, 1 = yes), reflecting this fundamental level of disease recognition.

- Smoking cessation

Smoking cessation was assessed among participants who were smokers at baseline. Smoking cessation was defined as abstinence from smoking at post-intervention or follow-up assessments, and responses were recorded as binary variables (0 = continued smoking, 1 = smoking cessation).

- Exercise adherence

Exercise adherence was assessed using the question, “Do you exercise regularly?” Responses were recorded as binary variables (0 = no, 1 = yes).

#### 2.6.2. Functional Status Outcomes

- Cognitive function

Cognitive function was evaluated using the Prescreening Korean Dementia Screening Questionnaire (KDSQ-P) [28]. The questionnaire consisted of five items comparing current cognitive abilities to those one year prior, including memory decline, difficulty performing activities of daily living, and difficulty recalling important events. Responses were rated on a 3-point scale (0 = seldom, 1 = sometimes, and 2 = often), resulting in total scores ranging from 0 to 10. Scores of four or higher indicated a need for further dementia screening. The reliability of this instrument, as measured by Cronbach’s α, was 0.885.

- Korean instrumental activities of daily living

Instrumental activities of daily living were assessed using the Korean version of the Instrumental Activities of Daily Living scale (K-IADL), the validity of which was established by Kang et al. [29]. The scale was administered to COPD patients discharged to their homes and consists of 11 items assessing functional performance over the past four weeks, including shopping, use of transportation, money management, housekeeping, meal preparation, medication management, and recent memory. Responses were rated on a 4-point Likert scale (0 = independent, 1 = needs some help, 2 = needs considerable help, and 3 = unable to perform independently), with total scores ranging from 0 to 33. Lower scores indicated better instrumental activities of daily living. The reliability of the K-IADL in this study, as measured by Cronbach’s α, was 0.817.

#### 2.6.3. Psychological Well-Being Outcomes

- Self-efficacy

Self-efficacy was evaluated using a seven-item scale developed by Pearlin et al. [30]. Sample items included statements such as, “I have little control over the things that happen to me,” “There is really no way I can solve some of the problems I have,” and “What happens to me mostly depends on me.” Responses were rated on a 5-point Likert scale (1 = strongly disagree to 5 = strongly agree). Negatively worded items were reverse-coded. The scale demonstrated high reliability, with a Cronbach’s α of 0.902.

- Hospital anxiety and depression

Anxiety and depression were assessed using the Hospital Anxiety and Depression Scale (HADS), developed by Zigmond and Snaith [31]. The HADS consists of 14 items, with seven odd-numbered items assessing anxiety and seven even-numbered items assessing depression. Each item is rated on a 4-point scale (0 = not at all, 1 = occasionally, 2 = quite often, and 3 = most of the time), yielding subscale scores ranging from 0 to 21. Positively worded items were reverse-coded so that higher scores indicated greater levels of anxiety and depression. The reliability of the instrument in this study, as measured by Cronbach’s α, was 0.872.

#### 2.6.4. Patient-Centered Outcomes

- Care transitions measure

Transitional care quality was assessed using the PACT-M scale [32,33]. The PACT-M1 subscale was used to evaluate discharge preparedness. This 9-item scale includes statements such as “Before leaving the hospital, I was confident I understood how to manage my medication” and “Before leaving the hospital, I felt confident about what to do if my health became worse at home.” Each item is rated on a 5-point Likert scale (1 = strongly disagree to 5 = strongly agree) [32]. Cronbach’s alpha for the PACT-M1 was 0.756.

Post-discharge self-management capacity was assessed using PACT-M2, which comprises eight items, such as “I feel confident about managing my health at home” and “I have the support I need from community health services (e.g., doctors, nurses, and home care staff).” Each item is rated on a 5-point Likert scale (1 = strongly disagree to 5 = strongly agree) [33]. Cronbach’s alpha for the PACT-M2 was 0.996.

- Patient satisfaction

Patient satisfaction was assessed using the Patient Admission Experience Questionnaire developed by the Korean Health Insurance Review & Assessment Service (HIRA), which was modified to suit the research context. This 10-item scale includes questions such as “Were your or your family’s/caregiver’s opinions reflected in the examination or treatment decision process?” and “Could you understand well what your healthcare professionals (doctors, nurses, etc.) explained about medical terminology, disease condition? Each item is rated on a 5-point scale (1 = strongly disagree to 5 = strongly agree) [34]. The reliability of this instrument, as measured by Cronbach’s α, was 0.926.

### 2.7. Data Collection

Data were collected from November 2022 to September 2024 from patients with COPD hospitalized at Kangwon National University Hospital, Hallym University Chuncheon Sacred Heart Hospital, and Seoul Boramae Medical Center, South Korea. Baseline data were obtained through face-to-face interviews with nurses. Post-intervention and follow-up data for the TCG were collected through nurse visits to the patients’ homes or nursing facilities. In the UCG, data were collected during outpatient visits. Post-intervention data were collected one month after discharge, and follow-up data were collected three months after discharge. Patient satisfaction and PACT-M1 were measured immediately before discharge, while PACT-M2 was assessed at one and three months post-discharge.

### 2.8. Data Analysis

Data analysis was performed using IBM SPSS Statistics version 26.0 (IBM Corp., Armonk, NY, USA) and R software (version 4.5.2; R Foundation for Statistical Computing, Vienna, Austria). Baseline characteristics of the TCG and UCG were summarized using descriptive statistics. Between-group comparability at baseline was examined using independent t-tests for continuous variables and chi-square tests or Fisher’s exact tests for categorical variables, as appropriate.

All primary analyses were conducted according to the intention-to-treat (ITT) principle, whereby all participants with baseline data were included in the analysis according to their originally assigned group, regardless of intervention adherence or follow-up completion.

Intervention effects over time for continuous outcomes, including cognitive function, K-IADL, self-efficacy, anxiety, and depression, were evaluated using linear mixed-effects models (LMMs) implemented in R. These models included fixed effects for group (TCG vs. UCG), time (baseline, 1 month, and 3 months), and the group-by-time interaction, along with a random intercept for each participant to account for within-subject correlation. Age was included as a continuous covariate in all models to adjust for baseline differences between groups. This approach allows inclusion of participants with incomplete follow-up data and accommodates missing outcome values under the assumption that data were missing at random (MAR), without requiring imputation.

For categorical outcomes, including disease awareness, smoking cessation rate, and exercise adherence, between-group differences at each time point were examined using chi-square tests in SPSS, and relative effect measures (risk ratios with 95% confidence intervals) were additionally calculated.

Patient-centered outcomes measured at a single time point, including discharge preparedness (PACT-M1), post-discharge self-management capacity (PACT-M2), and patient satisfaction, were analyzed using analysis of covariance (ANCOVA), with group as the independent variable and age included as a covariate.

Clinical outcomes such as all-cause and COPD-related readmissions, emergency department visits, and acute exacerbations were assessed as part of the overall trial but are reported in a separate manuscript currently under review. Accordingly, the present analysis focused on patient-centered, self-management–related sensitive outcomes. All statistical tests were two-sided, and a *p*-value of <0.05 was considered statistically significant.

## 3. Results

### 3.1. Homogeneity Test

Patient data were analyzed according to the intention-to-treat principle, with participants analyzed according to their originally assigned groups. The baseline characteristics of the study participants are summarized in Table 1. Most participants were male (83.7%), and the mean age was 76.25 years in the TCG and 78.14 years in the UCG. With respect to household composition, most participants in both groups lived as couples, and 92.3% were discharged to their homes. Regarding insurance coverage, 85.6% of participants were enrolled in the National Health Insurance program. In terms of healthcare expenditure, 42.2% reported experiencing a slight financial burden. Regarding lifestyle habits, 43.9% of participants were former drinkers and 50.5% were former smokers, with no significant differences between the TCG and the UCG.

### 3.2. Effects of the Transitional Care Service on Health-Related Self-Management Behaviors Outcomes

The effects of the TCSs on health-related self-management behaviors are presented in Table 2. For categorical self-management outcomes, disease awareness and exercise adherence did not differ significantly between the TCG and the UCG at baseline (disease awareness: 27.9% vs. 28.0%, RR = 1.00, 95% CI: 0.74–1.33; exercise adherence: 46.8% vs. 43.1%, RR = 1.09, 95% CI: 0.89–1.33). However, disease awareness was significantly higher in the TCG than in the UCG at both 1 month (83.5% vs. 33.6%, RR = 2.48, 95% CI: 2.04–3.02) and 3 months (94.3% vs. 35.7%, RR = 2.64, 95% CI: 2.19–3.18) (all *p* < 0.001). Similarly, exercise adherence was significantly higher in the TCG at 1 month (74.3% vs. 45.8%, RR = 1.62, 95% CI: 1.38–1.91) and 3 months (76.3% vs. 43.0%, RR = 1.78, 95% CI: 1.49–2.11) compared with the UCG (all *p* < 0.001). Among participants who were current smokers at baseline, smoking cessation rates were higher in the TCG at both 1 month (50.0% vs. 38.2%, RR = 1.31, 95% CI: 0.86–1.99) and 3 months (51.6% vs. 38.2%, RR = 1.42, 95% CI: 0.93–2.16); however, these between-group differences did not reach statistical significance (*p* = 0.27 and *p* = 0.14, respectively).

### 3.3. Effects of the Transitional Care Service on Functional Status Outcomes

The effects of the TCSs on functional status outcomes are summarized in Table 3. Linear mixed-effects models showed significantly smaller declines in cognitive function in the TCG compared with the UCG at both 1 month and 3 months (between-group differences: −0.81 (SE = 0.22) and −0.92 (SE = 0.22), respectively), with moderate to large effect sizes (SMDs = −0.70 and −0.80). IADL scores improved in the TCG but declined in the UCG, resulting in significant between-group differences at 1 month (−1.36 (SE = 0.51)) and 3 months (−1.77 (SE = 0.51)). The corresponding effect sizes ranged from small to moderate (SMDs = −0.46 and −0.60), indicating a clinically meaningful preservation of functional independence in the TCG.

### 3.4. Effects of the Transitional Care Service on Psychological Well-Being Outcomes

The effects of the TCS on psychological well-being outcomes are presented in Table 3. Self-efficacy scores declined from baseline in both groups, with a significantly greater decline observed in the UCG than in the TCG at both 1 month and 3 months (between-group differences: 2.15 (SE = 0.75) and 2.65 (SE = 0.75), respectively), corresponding to moderate effect sizes (SMDs = 0.43 and 0.53).

Anxiety scores decreased significantly more in the TCG than in the UCG at both time points (−1.36 (SE = 0.22) at 1 month; −1.45 (SE = 0.22) at 3 months), with moderate to large effect sizes (SMDs = −0.85 and −0.91).

Depression scores also declined in both groups but showed significantly greater reductions in the TCG, with between-group differences of −1.98 (SE = 0.34) at 1 month and −2.72 (SE = 0.34) at 3 months, and large effect sizes (SMDs = −0.91 and −1.25).

### 3.5. Patient-Centered Outcomes at Discharge

The results of the patient-centeredness outcomes are presented in Table 4. After adjusting for age, the PACT-M1 score was significantly higher in the TCG than in the UCG (adjusted mean difference = 3.25, *p* < 0.001), indicating greater discharge preparedness in the TCG. However, there was no significant difference in patient satisfaction between the groups (adjusted mean difference = 0.66, *p* = 0.21).

The post-discharge self-management capacity (PACT-M2) subscale score assessed during the follow-up visit was also significantly higher in the TCG than in the UCG (adjusted mean difference = 4.93, *p* < 0.001).

## 4. Discussion

As chronic diseases increasingly affect all age groups and are no longer associated with older age alone, discussions on effective management strategies have gained significant attention. In this study, nurses were trained in discharge management methods for patients with COPD. A patient-centered TCS was implemented by trained nurses, and its impact on health behaviors and patient-centeredness among patients with COPD was evaluated. The results support the following implications.

First, the nurse-coordinated TCS was effective in improving disease awareness and exercise adherence. These findings are consistent with the results of our interim analysis, which demonstrated improvements in disease awareness [35], and are also supported by previous studies reporting that transitional care services improve physical activity among patients with chronic conditions [8]. Accurate recognition of one’s disease extends beyond simple information acquisition; it constitutes a prerequisite for understanding appropriate treatment pathways and for engaging more actively in interactions with healthcare professionals by asking informed questions and participating in decision-making. These improvements may be attributed to the repeated delivery of self-management education during hospitalization and after discharge in this study. Patients with COPD often avoid physical activity and exercise due to dyspnea, fatigue, and activity limitations experienced in daily life. The self-management education provided during hospitalization and after discharge not only facilitated patients’ understanding of their disease but also reduced perceived barriers to exercise by offering concrete, feasible strategies—such as simple stretching and breathing exercises that could be practiced in daily life—thereby contributing to improved exercise adherence. Although exercise adherence increased significantly in the TCG, smoking cessation rates did not reach statistical significance at 3 months. This discrepancy highlights the distinct nature of these two health behaviors: whereas exercise adherence can be modified relatively quickly through goal setting and behavioral coaching, smoking cessation represents a more complex behavior that requires overcoming physiological nicotine dependence and may necessitate pharmacological interventions in addition to behavioral support. Nevertheless, the significant improvement in exercise adherence indicates that nurse-coordinated TCS can exert substantial effects on modifiable lifestyle behaviors that are largely under patients’ immediate volitional control. These findings further suggest that self-management education delivered as a one-time explanation at admission or discharge is insufficient for the internalization of disease understanding and behavioral change, and that repeated, stepwise education throughout the transitional period is essential for strengthening disease awareness and building a foundation for long-term self-management capacity.

Second, a critical finding of this study is that TCS demonstrated a protective effect against the functional decline commonly observed after discharge. Older adults hospitalized for chronic conditions frequently experience post-hospital syndrome, characterized by deterioration in cognitive and physical function that is not directly attributable to the primary illness [36]. Previous studies have reported that even a single hospitalization is associated with an approximately 2.4-times faster rate of cognitive decline, and that cognitive impairment substantially increases the likelihood of subsequent declines in physical function, including mobility [37,38]. Consistent with this trajectory, the UCG in the present study exhibited declines in both cognitive function and instrumental activities of daily living. In contrast, the TCG experienced significantly less cognitive decline and demonstrated improvements in functional independence. These findings suggest that extending care beyond the hospital setting into patients’ home environments enabled nurses to directly identify and mitigate real-world barriers to independent living—such as difficulties with medication management and fall risks. In this way, TCS incorporating home visits may have functioned as a critical buffer against post-discharge frailty and functional deterioration. Given that cognitive function and IADL are key indicators of autonomy in community living—encompassing medication use, mobility, and problem-solving in daily life—these results indicate that TCS has the potential to reduce care gaps after discharge and to lower the risk of functional decline–driven admissions to nursing hospitals or long-term care facilities. Accordingly, TCS that include home visits by nurses and multidisciplinary teams should be considered a core practical strategy for continuously monitoring and promptly adjusting care in response to functional changes in patients’ real-life contexts following discharge.

Third, although self-efficacy declined over time in both groups, the decline was significantly greater in the UCG than in the TCG. In contrast, patients in the TCG maintained relatively stable levels of self-efficacy. In addition, the TCG showed significant reductions in anxiety and depressive symptoms. These findings suggest that weekly telephone monitoring conducted by nurses during the first month after discharge played a central role in promoting emotional stability and mitigating the psychological burden associated with disease management. Patients with COPD frequently experience anxiety and depression due to dyspnea, fear of symptom exacerbation, and repeated hospitalizations; in patients with more severe disease, such emotional distress may further restrict daily activities [39]. The present findings underscore the importance of post-discharge interventions that continue to monitor patients’ emotional states and enable early identification of anxiety and depression, followed by appropriate support and counseling. In addition, telephone monitoring allowed nurses to collaboratively review challenges encountered during the self-management process and to assist patients in setting realistic and achievable goals within their home environments.

Fourth, Patients with COPD in the TCG showed higher scores for discharge preparedness and post-discharge management capacity than those in the UCG. This suggests that nurses provided accurate and relevant information on medications, health management, and strategies for managing acute exacerbations. Communication between nurses and patients is not a unidirectional transfer of instructions but a reciprocal interaction where opinions are exchanged. To support this, systematic training programs should be developed to equip nurses with the skills to effectively deliver the information that patients need and to foster meaningful communication. These programs should enhance nurses’ communication skills by considering factors such as their competence, experience, and communication settings (e.g., face-to-face, phone, and home visits). Additionally, they should provide resources and strategies tailored to patient characteristics, such as disease status and educational level, and address the needs of patients facing barriers to language and comprehension.

Overall, this study highlights the structural role of nurses as key coordinators within transitional care. By bridging gaps between hospital and community-based care, nurse-coordinated TCS may contribute to improved continuity of care and more efficient use of healthcare resources. These findings have important implications for healthcare policy and the design of integrated care systems.

This study had several limitations. First, although the intervention was delivered by a relatively small number of trained nurses, this may have enhanced intervention fidelity and consistency; however, it may limit the generalizability of the findings to broader clinical settings. Second, this study primarily examined the short-term effects of TCS; therefore, the long-term impact of the intervention on health behavioral outcomes remains unclear. Third, although participants received standardized in-hospital education, variations in the length of hospital stay were not fully accounted for, which may have influenced the overall exposure to the intervention and should be considered when interpreting the findings. Fourth, although clinical severity was controlled through stratified randomization based on FEV1, factors such as prior hospitalization frequency that may influence patients’ self-management capacity were not fully controlled at baseline. Finally, the patient-centered indicators used in this study—discharge preparedness, post-discharge self-management capacity, and patient satisfaction—may not fully capture the multidimensional nature of patient-centered outcomes.

Future studies should evaluate the scalability and generalizability of the intervention by incorporating a multidisciplinary approach involving not only nurses but also social workers, physicians, and community-based healthcare providers. In addition, the length of hospital stay should be considered as a potential confounding factor, and factors such as prior hospitalization experience that may influence patients’ self-management capacity should be controlled at baseline. Furthermore, more comprehensive indicators should be included to better capture multidimensional patient-centered outcomes.

## 5. Conclusions

A transitional care service delivered by nurses trained in discharge management effectively improved health-related self-management behaviors (disease awareness, smoking status, and exercise adherence), functional status (cognitive function, and instrumental activities of daily living), psychological well-being (self-efficacy and reduced anxiety and depressive symptoms), and patient-centered outcomes (discharge preparedness and post-discharge management) among patients with COPD. These findings provide valuable foundational evidence for the development of patient-centered transitional care services for individuals with chronic diseases, underscoring the importance of tailored and continuous care strategies throughout the post-discharge period. Notably, nurse-coordinated TCS offers key advantages, including enhanced continuity of care, individualized patient support, and effective coordination between healthcare institutions and community-based organizations. Given these strengths, TCS has strong potential to be implemented as a scalable and sustainable intervention within healthcare systems to improve outcomes for patients with chronic conditions. Future efforts should focus on integrating TCS into routine clinical practice and evaluating its long-term effectiveness across diverse healthcare settings and populations.

## Figures and Tables

**Table 1:** Baseline participant characteristics.

Variable		Total = 465		Between-Group Comparison				
				Transitional Care Group = 233		Usual Care Group = 232		*p* Value
		*n*	%	*n*	%	*n*	%	
Sex	Male	389	83.7	198	85.0	191	82.3	0.44
	Female	76	16.3	35	15.0	41	17.7	
Age (years), mean (standard deviation)		77.20 (8.63)		76.25 (8.56)		78.14 (8.61)		0.01
Household composition	One-person household	117	25.2	49	21.0	68	29.3	0.06
	Married couple	244	52.5	129	55.4	115	49.6	
	Living with children	81	17.4	40	17.2	41	17.7	
	Others	23	4.9	15	6.4	8	3.4	
Destination after discharge	Home	420	92.3	216	93.9	204	90.7	0.25
	Transfer to another hospital	28	6.2	10	4.3	18	8.0	
	Others	7	1.5	4	1.8	3	1.3	
Insurance type	Health insurance	398	85.6	204	87.6	194	83.6	0.17
	Healthcare aid—class	65	14.0	29	11.2	36	15.5	
	Others	2	0.4	-	1.2	2	0.9	
Financial burden due to healthcare expenditure	No burden at all	9	1.9	4	1.7	5	2.2	0.50
	Minimal	96	20.7	54	23.3	42	18.1	
	Average	103	22.2	54	23.3	49	21.1	
	Some	196	42.2	92	39.7	104	44.8	
	Heavy	60	12.9	28	12.0	32	13.8	
Drinking status	Non-drinker	163	35.1	74	31.8	89	38.4	0.14
	Former	204	43.9	106	45.5	98	42.2	
	Current	98	21.1	53	22.7	45	19.4	
Smoking status	Non-smoker	103	22.2	41	17.6	62	26.7	0.08
	Former	235	50.5	126	54.1	109	47.0	
	Current	127	27.3	66	28.3	61	26.3	

**Table 2:** Chi-Square Test Results for Disease Awareness, Exercise Adherence, and Smoking Cessation.

Outcomes by Month		Transitional Care Group (*n*, %)	Usual Care Group (*n*, %)	χ^2^ (*p*-Value)	RR (95% CI)
Disease awareness	Baseline	65 (27.9)	65 (28.0)	0.001 (0.97)	1.00 (95% CI: 0.74–1.33)
	1 mo	182 (83.5)	72 (33.6)	110.734 (<0.001)	2.48 (95% CI: 2.04–3.02)
	3 mo	199 (94.3)	74 (35.7)	158.183 (<0.001)	2.64 (95% CI: 2.19–3.18)
Exercise adherence	Baseline	109 (46.8)	100 (43.1)	0.635 (0.42)	1.09 (95% CI: 0.89–1.33)
	1 mo	162 (74.3)	98 (45.8)	36.650 (<0.001)	1.62 (95% CI: 1.38–1.91)
	3 mo	161 (76.3)	89 (43.0)	48.226 (<0.001)	1.78 (95% CI: 1.49–2.11)
Smoking cessation rate *	1 mo	32 (50.0)	21 (38.2)	1.23 (0.27)	1.31 (95% CI: 0.86–1.99)
	3 mo	33 (51.6)	21 (38.2)	2.19 (0.14)	1.42 (95% CI: 0.93–2.16)

RR, relative risk; CI, confidence interval; mo, months. * Smoking cessation rates were calculated among baseline smokers only, following the intention-to-treat principle.

**Table 3:** Linear Mixed-Effects Model Results for Functional Status and Psychological well-being Outcomes.

Outcomes by Month		Transitional Care Group		Usual Care Group		Between-Group Difference in Change from Baseline	
		Mean (SE)	Mean (SE) Change from Baseline	Mean (SE)	Mean (SE) Change from Baseline	Mean (SE)	Standardized Mean Difference (95% CI)
Functional Status Outcomes							
Cognitive function	Baseline	2.20 (0.15)	NA	2.70 (0.15)	NA	−0.49 (0.22) *	−0.42 (−0.79 to −0.05)
	1 mo	1.78 (0.15)	−0.43 (0.11)	2.59 (0.16)	−0.10 (0.11)	−0.81 (0.22) **	−0.70 (−1.07 to −0.33)
	3 mo	1.59 (0.16)	−0.61 (0.11)	2.51 (0.16)	−0.18 (0.11)	−0.92 (0.22) ***	−0.80 (−1.17 to −0.43)
IADL	Baseline	3.69 (0.36)	NA	4.14 (0.36)	NA	−0.46 (0.51)	−0.15 (−0.49 to 0.18)
	1 mo	3.29 (0.36)	−0.40 (0.28)	4.65 (0.36)	0.51 (0.28)	−1.36 (0.51) ***	−0.46 (−0.79 to −0.12)
	3 mo	3.06 (0.36)	−0.63 (0.28)	4.83 (0.36)	0.69 (0.28)	−1.77 (0.51) ***	−0.60 (−0.93 to −0.26)
Psychological well-being Outcomes							
Self-efficacy	Baseline	27.91(0.53)	NA	27.39 (0.53)	NA	0.64 (0.75)	0.13 (−0.17 to 0.42)
	1 mo	27.12 (0.53)	−0.83 (0.47)	24.94 (0.53)	−2.35 (0.47)	2.15 (0.75) **	0.43 (0.14 to 0.72)
	3 mo	26.64 (0.53)	−1.31 (0.47)	23.97 (0.53)	−3.32 (0.47)	2.65 (0.75) ***	0.53 (0.23 to 0.82)
Anxiety	Baseline	2.56 (0.16)	NA	3.06 (0.16)	NA	−0.50 (0.22) *	−0.31 (−0.59 to −0.04)
	1 mo	1.43 (0.16)	−1.14 (0.15)	2.79 (0.16)	−0.28 (0.15)	−1.36 (0.22) ***	−0.85 (−1.13 to −0.58)
	3 mo	1.20 (0.16)	−1.36 (0.15)	2.65 (0.16)	−0.41 (0.15)	−1.45 (0.22) ***	−0.91 (−1.18 to −0.63)
Depression	Baseline	5.74 (0.24)	NA	6.59 (0.24)	NA	−0.85 (0.34) *	−0.39 (−0.69 to −0.08)
	1 mo	4.20 (0.24)	−1.54 (0.20)	6.18 (0.24)	−0.41 (0.20)	−1.98 (0.34) ***	−0.91 (−1.22 to −0.61)
	3 mo	3.44 (0.24)	−2.31 (0.20)	6.16 (0.24)	−0.43 (0.20)	−2.72 (0.34) ***	−1.25 (−1.56 to −0.95)

SE, Standard Error; CI, Confidence Interval; NA, Not Applicable; mo, Month; IADL, Instrumental Activities of Daily Living. Values are estimated marginal means adjusted for age using linear mixed-effects models. * *p* < 0.05, ** *p* < 0.01, *** *p* < 0.001.

**Table 4:** Patient-centeredness outcomes: between-group comparisons.

Variable	Transitional Care Group		Usual Care Group		Between-Group Comparison (Adjusted)
	Baseline (T1)	3 mo (T2)	Baseline (U1)	3 mo (U2)	
	Mean (SD)	Mean (SD)	Mean (SD)	Mean (SD)	
Discharge preparedness	35.44 (6.01)	-	32.01 (5.82)	-	3.25 (<0.001)
Patient satisfaction	39.35 (5.50)	-	38.52 (5.65)	-	0.66 (0.21)
Post-discharge management	-	34.96 (4.86)	-	29.95 (4.39)	4.93 (<0.001)

T1, baseline value of the Transitional Care Group; T2, value at 3 mo in the Transitional Care Group; U1, baseline value of the Usual Care Group; U2, value at 3 mo in the Usual Care Group; mo, months; SD, standard deviation; -, not applicable.

## Data Availability

The datasets used in this study are available from the Clinical Research Data Repository of the National Evidence-based Healthcare Collaborating Agency (NECA) upon reasonable request and approval, via https://repository.neca.re.kr/data/37962192-5a7e-4259-8859-352f3c77cfb5 (accessed on 13 May 2026).

## Supplementary Materials

The following supporting information can be downloaded at: https://www.mdpi.com/article/10.3390/healthcare14101365/s1, File S1: CONSORT 2025 checklist of information to include when reporting a randomised trial [40].

## Abbreviations

The following abbreviations are used in this manuscript:

COPD	Chronic Obstructive Pulmonary Disease
ICD	International Classification of Diseases
ITT	Intention To Treat
K-IADL	Korean Instrumental Activities of Daily Living
PACT	Partners at Care Transitions Measure
TCG	Transitional Care Group
TCS	Transitional Care Service
UCG	Usual Care Group
SE	Standard Error
RR	Relative Risk
CI	Confidence Interval
